# A Locally Developed Electronic Health Platform in Uganda: Development and Implementation of Stre@mline

**DOI:** 10.2196/formative.9658

**Published:** 2018-09-24

**Authors:** Li Liang, Matthew O Wiens, Phaisal Lubega, Ian Spillman, Samuel Mugisha

**Affiliations:** 1 Faculty of Medicine University of Toronto Toronto, ON Canada; 2 University of British Columbia Vancouver, BC Canada; 3 Mbarara University of Science and Technology Mbarara Uganda; 4 Kisiizi Hospital Kisiizi Uganda; 5 Innovation Streams Limited Mbarara Uganda

**Keywords:** electronic health record, locally developed technology, appropriate technology, eHealth in low-resource settings

## Abstract

**Background:**

Electronic health records (EHRs) are especially important in low-resource settings due to their potential to address unique challenges such as a high number of patients requiring long-term treatments who are lost to follow-up, the frequent shortages of essential drugs, poor maintenance and storage of records, and inefficient clinical triaging. However, there is a lack of affordable and practical EHR solutions. Stre@mline is an EHR platform that has been locally developed by Ugandan clinicians and engineers in Southwestern Uganda. It is tailored to the specific context and needs of low-resource hospitals. It operates without internet access, incorporates locally relevant standards and key patient safety features, has a medication inventory management component, has local technical support available, and is economically sustainable without funding from international donors. Stre@mline is currently used by over 60,000 patients at 2 hospitals, with plans to expand across Uganda.

**Objective:**

The purpose of this article is to describe the key opportunities and challenges in EHR development in sub-Saharan Africa and to summarize the development and implementation of a “Made-for-Africa” EHR, Stre@mline, and how it has led to improved care for over 60,000 vulnerable patients in a rural region of Southwestern Uganda.

**Methods:**

A quantitative user survey consisting of a set of 33 questions on usability and performance was conducted at Kisiizi Hospital. Users responded to each question through a Likert scale with the values of strongly disagree, disagree, agree, and strongly agree. Through purposive sampling, 30 users were identified and 28 users completed the survey.

**Results:**

We found that users were generally very satisfied with the ease of use of Stre@mline, with 96% (27/28) finding it easy to learn and 100% (28/28) finding it easy to use. Users found that Stre@mline was helpful in improving both clinical efficiency and enhancing patient care.

**Conclusions:**

The partnership of local clinicians and developers is crucial to the design and adoption of user-centered technologies tailored to the specific needs of low-resource settings. The EHR described here could serve as a model for the development of future technologies suitable for developing countries.

## Introduction

### Background

Although often viewed as a resource only available to well-funded health facilities in developed countries, electronic health records (EHRs) are especially important for low-resource settings such as those in sub-Saharan Africa due to the unique challenges they can address. A well-developed EHR system can fill critical gaps common to such environments, such as assisting busy frontline workers to treat diseases, especially those requiring long-term treatment and monitoring (such as HIV and diabetes), and to anticipate and prevent frequently reported shortages of essential drugs. Furthermore, limited human resource capacity often leads to poorly developed and maintained medical records as well as poor triaging of patients presenting to hospitals. An optimized EHR can efficiently help address these ubiquitous issues. While EHRs can, and should, be instrumental in helping resource-poor countries meet the UN Sustainable Goals, many unique challenges have prevented the development and scaling of EHR systems in many resource-poor environments, including Uganda. The purpose of this article is to describe the key opportunities and challenges in EHR development in sub-Saharan Africa and to summarize the development and implementation of a “Made-for-Africa” EHR and how it has led to improved care for over 60,000 vulnerable patients in a rural region of Southwestern Uganda.

### Opportunities and Challenges With Electronic Health Records

One of the biggest public health challenges to the success of managing diseases like HIV/AIDS and tuberculosis is the high rates of patients who are “lost to follow-up” [1]. EHRs have been proposed as one of the top solutions [2,3], with preliminary evidence suggesting EHRs can significantly reduce the estimated 24% of all HIV patients lost to follow-up [4]. In addition, EHRs are useful for the long-term follow-up of chronic illnesses like cancer, high blood pressure, and diabetes and to ensure that patients are treated following the correct guidelines. Moreover, accurate and thorough data collection using EHRs can also inform targeted allocation of limited resources by recognizing trends and helping define priorities.

Inventory management and triaging challenges are also especially important for low-resource settings. Severe shortage of essential drugs as well as the dispensing of expired drugs are the major problems faced by patients in developing countries [5,6]. Poor patient record storage as a result of staffing shortages is another key challenge in low-resource hospitals. Thus, EHRs can play a significant role in allowing hospitals to better track and plan resources to ensure that essential medications are in stock and not expired and in ensuring that medical records are available and organized. Both of these are expected to result in improved patient care. Another intriguing advantage offered by EHRs is that supportive tools and guidelines for triaging, which are especially important given severe staffing shortages in low-resource settings [6], can be embedded within EHRs to focus resources on the most vulnerable patients. Improved triaging has been demonstrated to improve outcomes, including mortality, without the need for additional staffing [7].

Despite the many potential advantages of EHR systems, several important implementation challenges specific to developing countries have been identified. High initial costs associated with computer purchase and infrastructure setup is a critical barrier [8]. However, this can, at least, be partially mitigated by potential cost saving from reductions in record-keeping costs [9] and significantly improved staff efficiency [10]. Arguably, the most critical challenge to the success of existing EHR systems is the lack of involvement of local staff in the design and testing of systems to ensure that they are designed according to local needs and workflows. Other significant challenges include the purchase cost of the EHR software, the lack of local and inexpensive technical support to maintain EHR systems, frequent power and internet outages, the lack of computer literacy, and the fact that most EHRs are not sustainable without funding from international partnerships [8,11,12]. Overcoming these challenges will be crucial to ensure successful implementation and scaling of any EHR in a low-resource setting.

### Stre@mline: A “Made-for-Africa” Electronic Health Record

#### Local Setting

In Uganda, there are currently 1.5 million people living with HIV/AIDS and 79,000 new cases of active tuberculosis are reported every year. In addition, as many as 50% of essential drugs are not available at public hospitals [13]. Moreover, there is a significant shortage of qualified health workers, with only 0.12 physicians and 1.3 nurses per 1000 people [14]. Uganda is one of the poorest countries in the world, with about one-third of Ugandans living on less than US $1.90 purchasing power parity per day. The region in which the described EHR was implemented (Kisiizi, a rural region in Southwestern Uganda) is among the poorest regions in Uganda, thus, providing an ideal context for the development of a system ideal for rural and remote health facilities in sub-Saharan Africa. The EHR system, Stre@mline, was codeveloped by Ugandan software developers at a technology startup named istreams (acronym for Innovation Streams) and a team of physicians from the Kisiizi Hospital, a private not-for-profit hospital in Southwestern Uganda.

#### Partnerships

The partnership between istreams and the Kisiizi Hospital was initiated in 2013 when physicians from the hospital approached istreams to assist in developing a very simple EHR to overcome several specific challenges faced by the hospital that could not be adequately addressed by commercially available software applications. Over the following 2 years, the team met regularly to develop and pilot a platform tailored to the specific constraints and criteria of a busy Ugandan hospital. The resulting product is a sustainable and scalable EHR system that can address many of the shortcomings in the existing EHR platforms described earlier. The development cost for Stre@mline was funded by a National Science & Technology Improvement Grant (NSTIP) from Uganda National Council for Science & Technology (UNCST) as well as from the Kisiizi Hospital.

#### Unique Features of Stre@mline

In addition to the standard features such as interfaces for clinicians from different departments to communicate with one another, Stre@mline has a pharmacy interface that can be seen by prescribers, and it continuously updates stock, expiry date, and price of all drugs in real time. Stre@mline enrolls the patients’ journey through a system of unique identifiers. If a patient’s folder name is not present in the system, a unique identifier specific to the hospital is generated for the patient (eg, KH-60570 for one patient and KH-60571 for the subsequent patient). In addition, identifier information including the patient’s first and last name, village name, phone number, sex, and age is also recorded in the database because oftentimes, there are multiple patients with the same name in the hospital’s database. For returning patients, at triage, a patient’s folder will be searched based on the abovementioned identifiers. When the patient’s folder is found, a new file will be created for the current visit; this file will be time stamped and appended to the patient’s previous files in the folder.

The Stre@mline platform is also unique as it does not require internet access to operate. This is very important as internet access is very unreliable and expensive in Uganda. Instead, the program operates through a local area network consisting of 30 computers connected with each other and a local central server run through Ethernet cables. All data are backed up on the central server that is backed up through a battery-powered inverter system designed to prevent data loss during power outages.

Another key sustainability feature of the platform is that istreams offers local technical support to promptly solve technical issues and make improvements according to user needs. Through continuous and consistent user input, Stre@mline incorporates many locally relevant standards and guidelines, such as the locally developed Kissizii Early Warning System and the World Health Organization Emergency Triage Assessment and Treatment guidelines, local drug guidelines, and built-in linkage to the Uganda National Drug Authority adverse drug reactions reporting system. These systems are described in more detail in Table 1. Codesigning with local clinicians ensures simplicity of training and general use. Clinicians who have basic knowledge of using a computer can learn the key features of Stre@mline in less than 30 minutes. Furthermore, Stre@mline can be customized to integrate with local insurance plans, such as the Kisiizi Hospital Health Insurance Scheme used by 40,000 users, to monitor health-seeking behaviors. Finally, Stre@mline is affordable, with funding for development of this program coming entirely from within Uganda.

Currently, Stre@mline has been implemented in 2 hospitals in Southwestern Uganda and has been used for over 60,000 patients. Furthermore, the system is being set up in 3 additional hospitals in the region. istreams hopes to expand the system to 50 hospitals in Uganda over the next 2 years.

**Table 1:** Key local health care challenges addressed by Stre@mline.

Issue and problem		Stre@mline response
**Follow-up for long-term treatments**		
	High number of patients lost to follow-up	Monitoring of follow-up attendance, facilitation of contacting patients to ensure good on-going care in place
**Medicines**		
	Severe shortages of drugs	Live monitoring of stock levels of medicines and triggering ordering in good time to avoid stock-outs
	Drugs often expire in storage, wasting valuable resources	Warns pharmacists of drugs due to expire in 2 months, facilitating better resource planning by pharmacists and prescribers
	Auditing of drug prescribing errors is often poor or erratic	Facilitates 100% capture of prescribing errors through built-in linkage to the Uganda National Drug Authority drug reactions reporting system
**Triage**		
	Triage often poorly done, especially in children	Incorporates the World Health Organization Emergency Triage, Assessment, and Treatment (ETAT) tool and the locally developed Kisiizi Early Warning System
	Paper-based triage systems were often omitted or only partially done	Ensures that 100% children are properly triaged using ETAT tool as it uses mandatory fields. Users rapidly learn the new routine and comply happily as they see the benefits.
**Medical records**		
	Often incomplete, poor quality records were kept	Captures key data relating to a patient’s symptoms, investigations, treatment, and follow-up
	Patients often forget to bring previous notes, images, etc, and may end up undergoing unnecessary duplicate tests	Allows files to be stored (eg, x-rays, clinical letters, and photographs, for immediate access in future)
**Customization**		
	Commercial systems are often difficult and expensive to customize to local requirements	Stre@mline is designed to allow free, easy, and comprehensive customization by local institutions to ensure that the system is optimal for the local environment

## Methods

A quantitative user survey was conducted at the Kisiizi Hospital by a Masters student at the Mbarara University of Science and Technology, who is not affiliated with istreams, between January and June 2017. It was a paper-based survey consisting of a set of 33 questions on system usability and performance. Users responded to each question through a Likert scale with the values of strongly disagree, disagree, agree, and strongly agree. Through purposive sampling, 30 users were identified to complete the survey. In total, 28 users (6 doctors, 3 clinical officers, 6 nurses, 4 other health professionals, 6 administrative staff, and 3 support staff) consented and completed the survey. The analysis of survey data was performed primarily through descriptive statistics using Microsoft Excel (Redmound, USA). The key findings are described below.

## Results

The results of this survey showed that users were generally very satisfied with the ease of use of Stre@mline, with 96% (27/28) finding Stre@mline easy to learn and 100% (28/28) finding it easy to use. The Stre@mline platform has addressed several problems at the hospital. First, Stre@mline has allowed physicians at the Kisiizi Hospital to reliably access patients’ past medical records and investigations, which was generally not possible with the prior paper-based system. This feature has also increased workflow efficiency, with 80% (8/20) of users agreeing that it has allowed them to “see more patients in a day” as well as increased apparent trust in physicians by patients (as identified by clinicians). The embedded guidelines and triage assistance within Stre@mline has also substantially improved patient care, with 100% (20/20) of respondents agreeing that it has improved their “decision making.”

Furthermore, clinicians interviewed at the Kisizi Hospital generally agreed that Stre@mline has allowed clinicians to prescribe alternatives if one drug is not in stock, and this has, in turn, *“improved patient care and compliance rates.”* Stre@mline has also improved resource planning by allowing pharmacists to track their drug stocks in real time, thus, improving consistent stocking and availability of essential medications at the hospital, with 96% of clinicians agreeing that it has *“provided better mechanism for drug availability”* and 100% agreeing that *system has led to “safer and more reliable prescriptions.”*

## Discussion

### Principal Findings

Designing the EHR according to the specific needs of Ugandan hospitals has been critical to the successful implementation of Stre@mline. Having senior clinical input throughout the process has ensured the achievement of maximum benefits for patient care. In contrast, many health information technology programs fail when the software use is difficult for the intended users, thus, reducing the efficiency of the provider. Thus, it is critical to understand the clinical workflows, the patient journey, and key clinical data points needed.

Partnerships between local technology entrepreneurs and clinicians have the potential to not only create well-designed, but more importantly, sustainable and scalable technological solutions for these settings. Such technologies are more likely to have access to effective local support for maintenance and further development through an intimate understanding of local needs. Furthermore, these solutions tend to be more economically sustainable, with less external donor funding needed. In addition, leveraging local pride can be an important contributor to the adoption of any technology and has certainly been well leveraged in the development and application of Stre@mline. Supporting local developers of health care technologies financially and technologically is, therefore, a model that may be far more sustainable and impactful than developing such technologies in Western countries. Finally, another key to the success of Stre@mline in Kisiizi has been strong leadership from clinician-administrators who ensured and mandated computer workshops for all hospital employees and organized piloting the EHR sequentially, one department at a time, until it was scaled across each department. The approach described could thereby serve as a model for the development of future appropriate technologies for research-limited settings.

### Limitations

Limitations of the current system include a lack of data portability between different hospitals because data are currently stored on local area networks. In addition, although the system is low cost, it may still be cost prohibitive for small public hospitals and clinics within Uganda and other African countries. Finally, quantitative data on cost savings and patient safety are yet to be collected and analyzed, limiting the ability to generate any cost metrics. These data are, however, currently being collected and will be incorporated into a cost-benefit analysis in the future.

### Conclusions

Stre@mline is a locally developed EHR system tailored to the specific needs of resource-constrained settings. It is unique in being entirely locally developed through a partnership between a local hospital and a local technology company; it has been developed and is sustainable without funding from outside Uganda. The EHR system is currently being used by over 60,000 patients at 2 hospitals across Uganda with plans for further scaling. The process described here could serve as a model for the development of future appropriate technologies in developing countries.

## Abbreviations

EHR: electronic health record
ETAT: Emergency Triage, Assessment, and Treatment
NSTIP: National Science & Technology Improvement Grant
UNCST: Uganda National Council for Science & Technology

## References

[1] Rosen S, Fox MP, Gill CJ (2007). Patient retention in antiretroviral therapy programs in sub-Saharan Africa: a systematic review. PLoS Med.

[2] Nucita A, Bernava GM, Bartolo M, Masi FDP, Giglio P, Peroni M, Pizzimenti G, Palombi L (2009). A global approach to the management of EMR (electronic medical records) of patients with HIV/AIDS in sub-Saharan Africa: the experience of DREAM software. BMC Med Inform Decis Mak.

[3] Forster M, Bailey C, Brinkhof M, Graber C, Boulle A, Spohr M, Balestre E, May M, Kleiser O, Jahn Andreas, Egger M (2008). Electronic medical record systems, data quality and loss to follow-up: survey of antiretroviral therapy programmes in resource-limited settings. Bull World Health Org.

[4] Fraser HSF, Allen C, Bailey C, Douglas G, Shin S, Blaya J (2007). Information systems for patient follow-up and chronic management of HIV and tuberculosis: a life-saving technology in resource-poor areas. J Med Internet Res.

[5] Linden AF, Sekidde FS, Galukande M, Knowlton LM, Chackungal S, McQueen KAK (2012). Challenges of surgery in developing countries: a survey of surgical and anesthesia capacity in Uganda's public hospitals. World J Surg.

[6] Blaya JA, Fraser HSF, Holt B (2010). E-health technologies show promise in developing countries. Health Aff (Millwood).

[7] Molyneux E, Ahmad S, Robertson A (2006). Improved triage and emergency care for children reduces inpatient mortality in a resource-constrained setting. Bull World Health Organ.

[8] Jawhari B, Ludwick D, Keenan L, Zakus D, Hayward R (2016). Benefits and challenges of EMR implementations in low resource settings: a state-of-the-art review. BMC Med Inform Decis Mak.

[9] Poissant Lise, Pereira Jennifer, Tamblyn Robyn, Kawasumi Yuko (2005). The impact of electronic health records on time efficiency of physicians and nurses: a systematic review. J Am Med Inform Assoc.

[10] Were MC, Emenyonu N, Achieng M, Shen C, Ssali J, Masaba JPM, Tierney WM (2010). Evaluating a scalable model for implementing electronic health records in resource-limited settings. J Am Med Inform Assoc.

[11] Fraser Hamish S F, Biondich Paul, Moodley Deshen, Choi Sharon, Mamlin Burke W, Szolovits Peter (2005). Implementing electronic medical record systems in developing countries. Inform Prim Care.

[12] Fritz F, Tilahun B, Dugas M (2015). Success criteria for electronic medical record implementations in low-resource settings: a systematic review. J Am Med Inform Assoc.

[13] Oluka P, Ssennoga F (2013). Tackling supply chain bottlenecks of essential drugs: A case of Uganda local government health units. 4th Int Procure Conf.

[14] (2017). World Bank Group.

